# Identification of fish source *Vibrio alginolyticus* and evaluation of its bacterial ghosts vaccine immune effects

**DOI:** 10.1002/mbo3.576

**Published:** 2018-01-19

**Authors:** Ji Cao, Jiajun Zhang, Lin Ma, Lin Li, Wenchang Zhang, Jinnian Li

**Affiliations:** ^1^ Key Laboratory of Zoonoses College of Animal Science and Technology Anhui Agricultural University Hefei China

**Keywords:** bacterial ghosts, identification, immune effects, *Vibrio alginolyticus*

## Abstract

*Vibrio alginolyticus* (*V. alginolyticus*) is a common pathogen for humans and marine aquatic animals. Vibriosis of marine aquatic animals, caused by *V. alginolyticus*, has become more prevalent globally in recent years. Hence, a safe and effective vaccine is urgently needed for the control of this disease. Here, the strain 16‐3 isolated from the large yellow croaker (*Larimichthys crocea*) suffered from canker was identified as *V. alginolyticus* based on morphological, biochemical, and 16S rDNA sequencing analysis. Then, recombinant temperature‐controlled lysis plasmid pBV220‐lysisE was electroporated into the strain 16‐3 to generate *V. alginolyticus* bacterial ghosts (VaBGs) by inducing lysis gene E expression, and the safety and immune effects of VaBGs were further investigated in mice and large yellow croaker. The results showed that VaBGs were as safe as formalin‐killed *V. alginolyticus* cells (FKC) to mice and fish. Compared with FKC and PBS groups, significant elevations of the serum agglutinating antibody titer, serum bactericidal activity, lymphocyte proliferative responses, and levels of four different cytokines (Th1 type: IL‐2, TNF‐α; Th2 type: IL‐4 and IL‐6) in serum were detected in the VaBGs group, indicating that a Th1/Th2‐mediated mixed immune response was elicited by the VaBGs. More importantly, after challenged with the parent strain 16‐3, VaBGs‐vaccinated mice and fish showed higher protection than FKC‐vaccinated mice, the relative percent of survival (RPS) being 60%, 66.7% and 40%, respectively. Taken together, this is the first demonstration that the newly constructed *V. alginolyticus* ghosts may be developed as a safe and effective vaccine against *V. alginolyticus* infection in aquaculture.

## 1 INTRODUCTION

*Vibrio alginolyticus* is one of the most common pathogenic marine Vibrio species and has been found to not only cause serious vibriosis in marine aquatic animals (Damir, Irena, Damir, & Emin, 2013; Gómezleón, Villamil, Lemos, Novoa, & Figueras, 2005; Kahlanakbi, Chaieb, & Bakhrouf, 2009; Sadok, Mejdi, Nourhen, & Amina, 2013), but also induce seafood‐poisoning or fatal extra‐intestinal infections in humans after consumption of raw or undercooked sea products (Lin, Ou, Dong, & Chen, 2001; Qiang, Qing, & Shen, 2006). Currently, antibiotics were mainly used in marine aquaculture to protect fish from *V. alginolyticus* infection. Nevertheless, the long‐term use of the antibiotics and chemotherapeutants lead to many negative impacts such as antibiotics residues and drug resistance, which drive us to find effective alternative means to control the *V. alginolyticus* infection in aquaculture.

Vaccination has become an effective means for preventing various infectious diseases in aquaculture industry. The reported *V. alginolyticus* vaccines for fisheries in laboratory studies mainly include formaldehyde‐killed vaccine, subunit vaccine, live‐attenuated vaccine, and naked DNA vaccine (Cai et al., 2010, 2013; Idris, Alhaj, Shamsudin, & Rahim, 2009; Li, Ma, & Woo, 2015). However, these vaccines have some disadvantages. Formaldehyde‐killed vaccines often result in a reduction in the ability of the vaccines to give full immunity by destroying the physical or chemical characteristics of components of bacterial surface structures. Subunit vaccines are often less immunogenic, and adjuvants have to be added to the vaccine formulation. Live‐attenuated vaccines have the risk of virulence reversion. The efficacy of naked DNA vaccine is low due to the degradation of DNA caused by the nucleases *in vivo*. Therefore, a safe and effective vaccine against *V. alginolyticus* is still needed. In recent years, bacterial ghosts (BGs) are empty and intact bacterial envelopes of Gram‐negative bacteria that are produced by controlled expression of the phage PhiX174 *lysisE* gene (Langemann et al., 2010). The resultant BGs retain the functional and antigenic determinants of the envelope with their living counterparts. Therefore, they possess good immunogenicity and adjuvant properties and can be used as a vaccine directly (Riedmann, Kyd, Cripps, & Lubitz, 2007). BGs vaccine can not only induce strong systemic and mucosal immune response in a similar way of living bacteria (Mayr et al., 2005; Riedmann et al., 2007; Wang & Lu, 2009), but also be produced in large quantities by simple fermentation without laborious purification procedures. In addition, BGs vaccine can be stored as freeze dried preparations at room temperature without the loss of efficacy for extended periods (Ra et al., 2009). As a result, BGs are suitable to be used as a new type of inactivated vaccine.

In our preliminary study, one pathogen, named the strain 16‐3, was isolated from the large yellow croaker (*Larimichthys crocea*) suffered from canker. The main objectives of the present study were (1) to identify the strain 16‐3, (2) to develop *V. alginolyticus* strain 16‐3 BGs vaccine, and (3) to evaluate the immune effects of the vaccine in mice and large yellow croaker.

## 2 MATERIALS AND METHODS

### 2.1 Ethics statements

Animal experiment was carried out in strict accordance with the recommendations in the Guide for the Care and Use of Laboratory Animals of the national laboratory animal welfare ethics, and protocols concerning animals were approved by the Ethical Committee of the Faculty of Veterinary Science of Anhui Agricultural University (Permit Number: 20130402). Every effort was made to reduce the number of animals used and minimize the suffering of the animals.

### 2.2 Bacterial strains and culture conditions

The strain 16‐3 was isolated from the large yellow croaker (*L. crocea*) with canker and was confirmed as the pathogen by experimental infection trials in fish (data not shown). Recombinant *Escherichia coli* DH5α harboring temperature‐controlled lysis plasmid pBV220‐lysisE was constructed by our laboratory. The strain 16‐3 was cultured in brain heart infusion broth containing 3% NaCl (BHI; Beijing Solarbio Science & Technology Co., Ltd., China) at 28°C, while recombinant *E. coli* DH5α was cultured in Luria Bertani medium (LB medium; Beijing Solarbio Science & Technology Co., Ltd., China) at 28°C.

### 2.3 Identification of the strain 16‐3

The strain 16‐3 was inoculated in BHI broth containing 3% NaCl and cultured for 24 hr at 28°C. Gram‐stained and phosphotungstic acid‐stained smears from cultures were examined by light microscopy and transmission electron microscope (H‐7700, Hitachi, Japan), respectively, to determine its morphology, structure, and dyeing property. Meanwhile, the pure cultures of the strain 16‐3 were inoculated on an ID32GN Gram‐negative identification strip (bioMerieux Sa, France) and cultured for 24 hr at 28°C. An ATB expression semi‐automatic analyzer (bioMerieux Sa, France) was used to measure its biochemical characteristics.

The 16S rDNA was amplified from the total genomic DNA of the strain 16‐3 by using universal primer pair consisted of the forward (5′‐AGAGTTTGATCCTGGCTCAG‐3′) and reverse (5′‐TACGGTTACCTTGTTACGACTT‐3′) oligonucleotides, then sequenced. After BLAST analysis with the measured sequence, 18 sequences with higher homology were selected. The phylogenetic tree was constructed by the adjacency method. Bootstrap analysis was used to measure the confidence level, and the bootstrap data set was 1,000 times.

### 2.4 Preparation of *V. alginolyticus* BGs vaccine

Temperature‐controlled lysis plasmid pBV220‐lysisE was extracted from recombinant *E. coli* DH5α (pBV220‐lysisE) using Plasmid DNA Extraction Kit (Omega, USA). The pBV220‐lysisE (3–4 μg) was mixed with the competent cell suspension of the strain 16‐3 (100 μl), transferred to a precooling 1 mm cuvette, and electroporated using a Gene Pulser (Bio‐Rad, CA, USA) and pulse controller (500 V, 25 μF, 200 Ω) producing a time constant of ±4.0 ms. After electroporation, 800 μl LB liquid broth immediately was added to the cuvette and incubated for 4 hr at 28°C. After incubation, cells were plated onto LB agar plates containing 100 μg/ml ampicillin and grown at 28°C for 24–36 hr. A single colony was picked and inoculated into LB liquid broth containing 100 μg/ml ampicillin with shaking overnight at 28°C. Then, the recombinant plasmid was extracted from the culture and identified by PCR and restriction enzyme digestion with *Eco*R I and *Pst* I.

The recombinant *V. alginolyticus* 16‐3 (pBV220‐lysisE) was added to 100 ml LB liquid broth containing 100 μg/ml ampicillin and incubated in a shaking bath at 28°C. When the culture reached OD_600_ of 0.3, the temperature was immediately upshift to 42°C to induce the expression of lysis gene E. Growth of bacteria in the culture was further monitored by measurement of OD_600_ every 30 min until no further decrease in the reading was observed. The resulting cell ghosts were then centrifuged at 12,000 × *g* for 10 min and washed three times with PBS, pH 7.2. Pellets were resuspended in saline followed by vacuum freeze drying for 24 hr under the condition without protective agent and then stored at 4°C for future use. The efficiency of *V. alginolyticus* BGs (VaBGs) formation was ascertained by determining the CFU counts of the culture prior to induction and of the freshly harvested ghosts on LB agar plates at 28°C for 3 days. The efficiency of ghost formation was calculated by the following formula: (1 − CFU counts of freshly harvested ghosts/CFU counts prior to induction) × 100%.

The lyophilized VaBGs were resuspended in sterile saline, and 100 μl of suspension was inoculated on LB agar plate in order to examine whether or not there were any living cells. In addition, samples for electron microscopy analysis were prepared according to previously described method (Chen, Li, & She, 2014). Then, the morphology and structure of VaBGs were observed using TEM (H‐7700, Hitachi, Japan) and SEM (S‐4800, Hitachi, Japan).

### 2.5 Preparation of formalin‐killed *V. alginolyticus* vaccine

*V. alginolyticus* strain 16‐3 was inoculated in 50 ml of BHI liquid broth containing 3% NaCl at 30°C for 24 hr before addition of formalin to the cultures to a final concentration of 0.3%. After a 24‐hr incubation, cells were pelleted and washed five times with PBS, resuspended in PBS to a bacterial concentration of 5 × 10^8 ^CFU/ml. Efficiency of inactivation was determined by plating 100 μl of the above bacterial suspension on to BHI agar plate and the presence of bacterial colonies was monitored for 3 days. The formalin‐killed whole cell vaccine (KFC) was frozen at 4°C before use.

### 2.6 Experimental animals and safety testing of *V. alginolyticus* BGs and KFC vaccine

Female BALB/c mice, weighing 20 ± 2 g, were purchased from the Experimental Animal Center of Anhui Medical University. The healthy large yellow croakers of 50 ± 5 g were obtained from a local fish farm. Experimental fish were transported to the laboratory and acclimated in seawater aquaria for 2 weeks prior to experiments. The fish were fed a commercial diet every day and starved for 1 day before and then during the experiment. Throughout the experimental period, water temperature was kept at 24–25°C and salinity was maintained constant at 26‰.

Safety evaluation was carried out to determine whether there were any adverse effects of vaccine on BALB/c mice and large yellow croakers prior to vaccination. Briefly, female mice were divided into two groups (10 mice per group) and injected intraperitoneally with five times the immune dose of VaBGs and KFC vaccine, respectively. Meanwhile, experimental fish (*n* = 10) were injected intraperitoneally with five times the immune dose of VaBGs. The general condition of the experimental animals were observed daily, and the mortality, visible adverse reactions at injection site were recorded. Necropsies were performed at 3 weeks postimmunization to monitor the lesions.

### 2.7 Immunization and challenge test

Female BALB/c mice (weight: 20 ± 2 g) were randomly divided into three groups: VaBGs group, FKC group and PBS control group, respectively, with 40 mice in each group. Two immunization groups were injected i.p. with VaBGs and FKC (10^8^ CFU per mouse), respectively, while the mice in the control group were injected with 0.3 ml of sterile PBS. After 2 weeks, a booster was given with the same immune dose. Meanwhile, healthy large yellow croakers (weight: 50 ± 5 g) were anesthetized with 0.02% of MS‐222 before vaccination, and randomly divided into two groups (40 fish in each group) including vaccinated group with VmBGs (10^8^ CFU per fish) and control group with 0.3 ml of sterile PBS. The same treatment was administered 2 weeks later (boosting).

On Day 28 postvaccination (dpv), totally 15 immunized mice or large yellow croakers in each group were i.p. challenged with 4.8 × 10^8 ^CFU/mouse or 1 × 10^8 ^CFU/fish *V. alginolyticus* strain 16‐3 (50 LD_50_ based on preliminary works). The animals were subsequently monitored the time of death for 14 days after challenge, and the protective efficacy of different vaccines was calculated as the relative percentage of survival (RPS) using the formula: (1 − % immunized mortality/% control mortality) × 100 (Amend,^1981^). To confirm that the deaths of immunized animals were caused by challenge, the challenge strain was re‐isolated from the liver of dead animals by streaking directly onto BHI agar plate and further confirmed by the API 20 NE system (BioMerieux, France) in accordance with the manufacturer's protocol.

### 2.8 Serum cytokine determination

At each time point (Days 7, 14, 21, and 28) of postimmunization, three mice were randomly taken from each group to draw orbital blood, and then blood samples were centrifuged to obtain serum for monitoring cytokine level. Double‐antibody sandwich ELISA assay was performed to determine serum levels of IL‐2 and TNF‐α as representative Th1 cytokines, and IL‐4 and IL‐6 as representative Th2 cytokines using ELISA kits (Calvin Biology Technology Co., LTD, Suzhou, China) according to the manufacturer's instructions.

### 2.9 Serum antibody titer determination and bactericidal activity assay

At each time point (Day 0, 7, 14, 21, 28, and 35) of postimmunization, serum was collected from three randomly selected experimental animals (BALB/c mice and large yellow croakers) in each group. Serum agglutination test was applied to reflect the titers of anti‐*V. alginolyticus* antibodies produced by immunized animals. Briefly, serial twofold dilution of test serum in PBS and equal volume of inactivated cell suspension of *V. alginolyticus* strain 16‐3 was added to each well of 96‐well microtitre plates. The plates were covered and incubated in humidified air at 25°C for 18 hr. The highest dilution of serum at which the serum was agglutinated with the antigen was the titer of anti‐serum and it was presented as log2 value of the dilution.

Serum bactericidal activity analysis was performed according to previously described method (Rao, Das, Jyotyrmayee, & Chakrabarti, 2006). Briefly, a volume of 500 μl *V. alginolyticus* 16‐3 suspension (1.93 × 10^8 ^CFU/ml) and 500 μl serum in each group were mixed in sterile eppendorf tubes. The mixture was incubated at 28°C for 1 hr, followed by plating on BHI agar plate containing 3% NaCl and incubating at 28°C for 48 hr. In the bacterial control group, the serum was replaced by PBS. The bactericidal activity was evaluated by the serum bactericidal rate using the following equation: serum bactericidal rate (%) = (1* − *the number of viable bacteria after immune serum treatment/the number of viable bacteria after PBS treatment) × 100%.

### 2.10 Spleen lymphocyte proliferation assay

Twenty‐eight days postimmunization, the proliferation activity of spleen lymphocytes was determined by MTT [3‐(4,5‐dimethylthiazol‐2‐yl)‐2,5‐diphenyltetrazolium bromide] method. Briefly, spleens from randomly selected three experimental animals (BALB/c mice and large yellow croakers) per group were ground into single cell suspension in Hank's solution, and spleen lymphocytes were separated using lymphocyte separation kit (Hao Yang Biotechnology Co. LTD, Tianjin). Cell viability was determined by trypan blue dye exclusion testing. A 100 μl of the lymphocyte suspension (5 × 10^6^ cells/ml) was added to RPMI‐1640 medium (containing 10% fetal calf serum, 2 mmol/L glutamine, 50 U/ml of penicillin, and 50 g/ml of streptomycin) and incubated in triplicate in 96‐well culture plates. T‐lymphocyte mitogen PHA (Sigma) was then added at a final concentration of 100 μg/ml. After cultured at 37°C for 72 hr, 10 μl of 5 mg/ml MTT (Sigma) was added into each well and incubated for another 4 hr. OD_570_ was measured after adding 100 μl DMSO (Sigma). The cell control well (without stimulation) and the blank control well (culture medium) were also measured. The results were presented as a stimulation index (SI), calculated as follows: SI = (OD_570 stimulated well_ − OD_570 blank well_)/(OD_570 unstimulated well_ − OD_570__blank well_).

### 2.11 Statistical analysis

All results were expressed as mean ± *SD*, one‐way ANOVA, and the Student–Newman–Keuls test were performed using SAS ver 6.12 software (SAS Institute Inc., Cary, NC, USA) for comparing significant differences among different groups. *p* value of <.05 was considered statistically significant.

## 3 RESULTS

### 3.1 Identification of strain 16‐3

**Figure 1:**
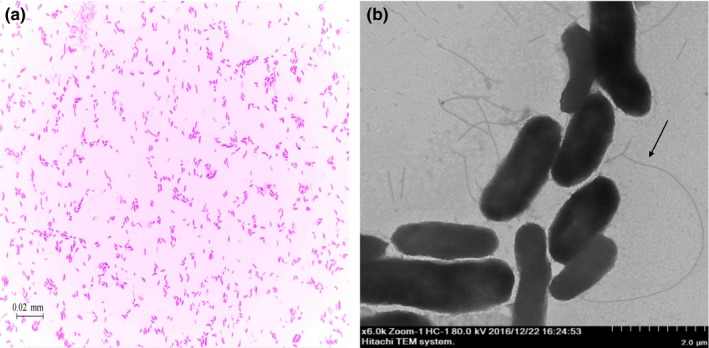
Morphology and structure of the strain 16‐3. (a) Light micrograph (1,000 × ). (b) Transmission electron micrograph (6,000 × ). Arrow shows flagellum

**Figure 2:**
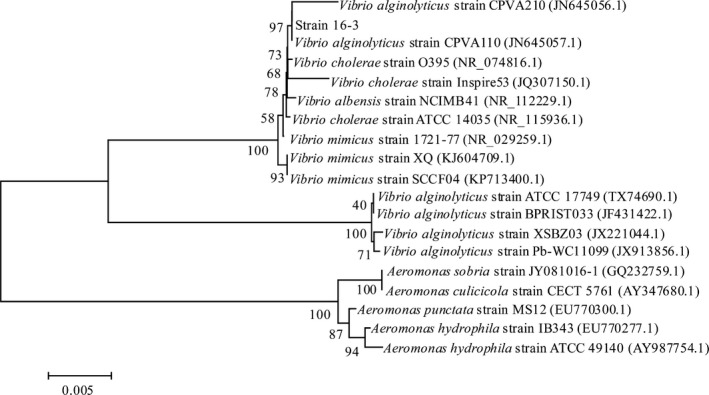
Phylogenetic tree based on 16S rRNA gene sequence of the strain 16‐3. The scale bar represents 0.005 substitutions per nucleotide position. The number at each branch points represents the bootstrap confidence value of the 1,000 repeated

As shown in Figure 1a, strain 16‐3 was Gram‐negative vibrio with a disordered orientation under the light microscope. TEM images revealed that the size of the bacterium was approximately (0.50~0.85) μm × (1.40~2.65) μm and that both ends of each bacterium were rounded with flagella (Figure 1b). As shown in Table 1, the biochemical characteristics of the strain 16‐3 were the same as the *V. alginolyticus* reference strain CPVA110 (GenBank accession number JN645057.1). Sequencing results demonstrated gene length of 16S rRNA from the strain 16‐3 was 1,508 bp with 99.6% and 98.4% homology with reference strains CPVA110 and CPVA210, respectively. The three strains naturally got together in the phylogenetic tree with 97% of bootstrap analysis confidence coefficient (Figure 2). Therefore, considering the phenotype and the 16S rRNA gene characteristics of the strain 16‐3, it could be confirmed as *V. alginolyticus*.

**Table 1:** Biochemical characteristics of the strain 16‐3 in comparison to the reference strain *Vibrio alginolyticus* CPVA110

Characteristics	Strain 16‐3	*V. alginolyticus* CPVA110	Characteristics	Strain 16‐3	*V. alginolyticus* CPVA110
L‐Rhamnose	+	+	Mannitol	+	+
Acetyl‐glucosamine	+	+	Glucose	+	+
D‐ribose	+	+	Salicin	−	−
Inositol	−	−	Melibiose	−	−
Sucrose	−	−	Fucose	−	−
Maltose	+	+	Sorbitol	−	−
Itaconate assimilation	−	−	Arabinose	−	−
Succinate assimilation	−	−	Malonate assimilation	−	−
Propionate assimilation	−	−	Caprate assimilation	−	−
Acetate assimilation	−	−	Valt assimilation	−	−
Lactate assimilation	+	+	Citrate	+	+
Alanine assimilation	−	−	Histidine assimilation	+	+
5‐keto gluconic acid	−	−	2‐keto gluconate	−	−
Glycogen	−	−	3‐hydroxy butyrate	−	−
3‐hydroxy benzoate	−	−	4‐hydroxy benzoate	−	−
Serine assimilation	−	−	Proline	+	+

+, 90% or more strains are positive; −, 90% or more strains are negative.

### 3.2 Identification of recombinant *V. alginolyticus* 16‐3 (pBV220‐lysisE)

**Figure 3:**
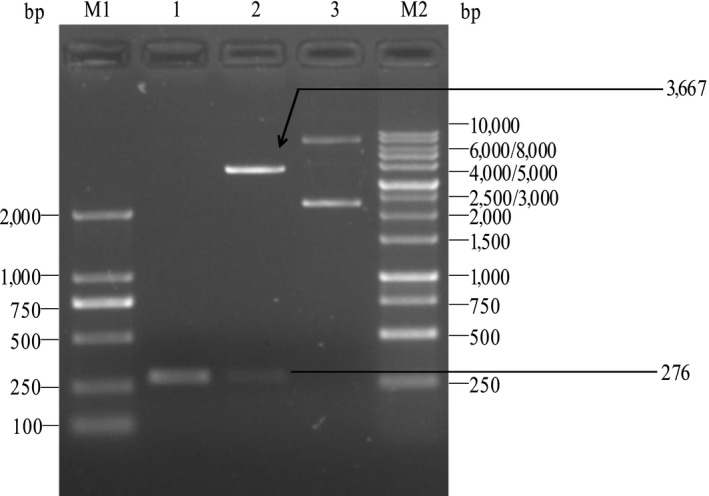
Identification of the recombinant *Vibrio alginolyticus* strain 16‐3 harboring plasmid pBV220‐lysisE by PCR and restriction enzyme digestion with *Eco*R I and *Pst* I. Lane M1: Marker DL2000, Lane1: PCR product of *lysis E* gene, Lane 2: Temperature‐controlled lysis plasmid pBV220‐lysisE double‐digested with *Eco*R I and *Pst* I, Lane 3: Temperature‐controlled lysis plasmid pBV220‐lysis E, Lane M2: Marker DL10000

The plasmid pBV220‐lysisE was electroporated into *V. alginolyticus* strain 16‐3 and identified by PCR and double‐enzyme digests. The gene *lysisE* (size in 276 bp) was amplified from the pBV220‐lysis E extracted from recombinant *V. alginolyticus* (Figure 3, lane 1). The *Eco*R I/*Pst* I double‐enzyme digests reaction showed one 3,667 bp DNA band and another 276 bp DNA band, which were same length with plasmid pBV220 and *lysisE* gene (Figure 3, lane 3). The results indicated that recombinant *V. alginolyticus* 16‐3 (pBV220‐lysisE) was successfully constructed.

### 3.3 Preparation and characterization of *V. alginolyticus* strain 16‐3 BGs

**Figure 4:**
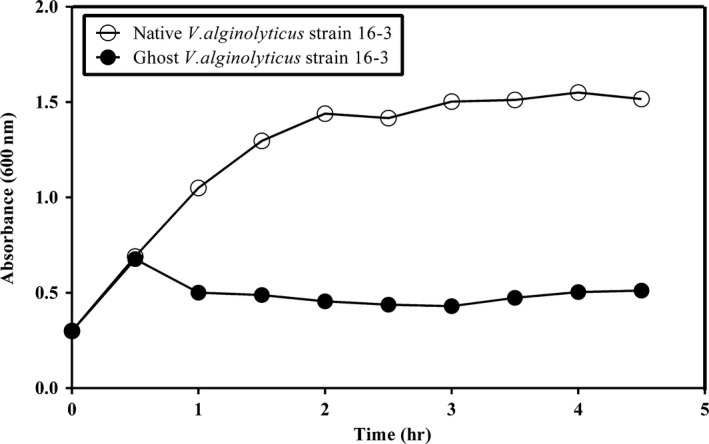
Growth and lysis curves of the recombinant *Vibrio alginolyticus* strain 16‐3 harboring plasmid pBV220‐lysis E by temperature induction of *lysis E* expression. At time zero, the cultures were shifted from 28 to 42°C

The *V. alginolyticus* strain 16‐3 BGs were generated by increasing the incubation temperature to 42°C. Recombinant *V. alginolyticus* 16‐3 (pBV220‐lysisE) lysis occurred at 30 min and was completed by 3 hr after induction (Figure 4). The efficiency of ghost induction in non‐lyophilized *V. alginolyticus* strain 16‐3 was about 99.91%. There was no bacterial growth on the BHI agar plate containing 3% NaCl after lyophilization.

**Figure 5:**
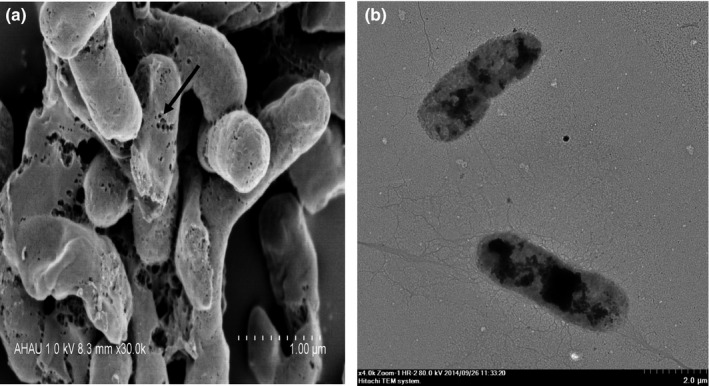
*Vibrio alginolyticus* strain 16‐3 BGs under electron microscopy. (a) Scanning electron micrograph (30,000 × ). Arrow indicates transmembrane tunnels. (b) Transmission electron micrograph (4,000 × ). The lysed cells show uneven and low electron density and retain the basic cell morphology

Transmembrane lysis pores ranging from 40.3 to 131.0 nm in diameter and shrinking cell surface were observed in *V. alginolyticus* strain 16‐3 BGs by scanning electron microscopy (Figure 5a). The structural integrity and the apparent loss of cytoplasmic materials were observed in *V. alginolyticus* strain 16‐3 BGs by transmission electron microscopy (Figure 5b). These results indicated that VaBGs possessed the normal array of cell surface antigens of live bacteria, but basically no cytoplasmic contents.

### 3.4 Safety of *V. alginolyticus* BGs and KFC vaccine

All experimental mice and fish that injected with five times the immune dose of *V. alginolyticus* BGs or KFC vaccine remained healthy without any symptoms, suggesting that these vaccines themselves were safe to these experimental animals.

### 3.5 Serum cytokines levels

**Figure 6:**
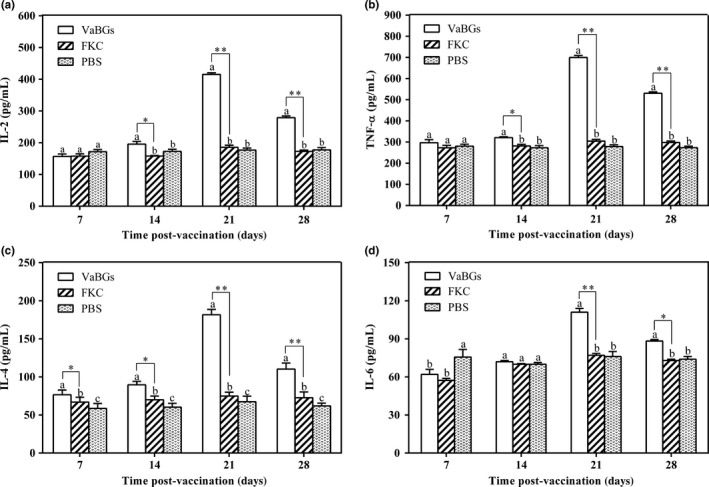
Serum cytokine levels in immunized mice detected by double‐antibody sandwich ELISA. Values presented as mean±*SD*,* n* = 3. Bars represent standard deviations. Different letters (a, b, c) on the bar indicate significant differences among different groups at the same time point. *p <* *.05, **p <* *.01 (VaBGs group versus FKC group)

To determine the immunologic mechanism induced by VaBGs, the levels of four different cytokines in the immunized mice serum at different time points postimmunization were detected with ELISA kits. As shown in Figure 6, except for 7 days after immunization, the Th1 type cytokine (IL‐2 and TNF‐ α) levels in the VaBGs group at other time points were significantly higher than those in the other two groups (FKC and PBS groups) (*p *<* *.05). However, there was no significant difference between the two groups (*p *>* *.05). The IL‐4 (Th2 type cytokine) levels in the two immune groups (VaBGs and FKC) at different time points were significantly higher than that in the PBS group (*p *<* *.05 or .01). Moreover, the IL‐4 level in the VaBGs group was significantly higher than that in the FKC group (*p *<* *.05). At 7 days after immunization, the IL‐6 (Th2 type cytokine) levels in the two immune groups were significantly lower than that in the PBS group (*p *<* *.05). At 14 days after immunization, there was no difference in the IL‐6 level among those three group (*p *>* *.05). At the other two time points, the IL‐6 level in the VaBGs group was significantly higher than those in the FKC and PBS groups (*p *<* *.05 or .01), but there was no statistical difference between the two groups (*p *>* *.05). These data indicated that VaBGs could elicit a mixed Th1/Th2 immunoresponse.

### 3.6 Serum agglutinating antibody titer and its bactericidal activity

**Figure 7:**
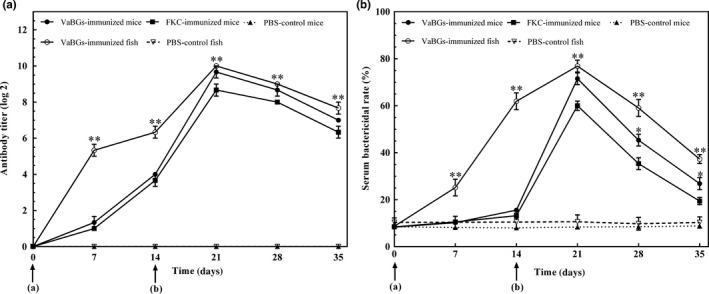
Serum agglutinating antibody titer against *Vibrio alginolyticus* and serum bactericidal activity of vaccinated animals at different time points postimmunization. (a) Serum agglutinating antibody titer. (b) Serum bactericidal activity. Data presented as mean ± *SD*,* n* = 3. *p <* *.05, **p <* *.01 (VaBGs group versus FKC or PBS group). a and b represent primary and booster immunization, respectively

As shown in Figure 7, the serum agglutinating antibody titer and its bactericidal rate in the immunized mice and fish gradually increased with the increase of immunization time and frequency. They all reached a peak at 21 days post immunization, with the mean geometric titer 9.67 log2 (VaBGs group in mice), 8.67 log2 (FKC group in mice), and 10 log2 (VaBGs group in fish), and the bactericidal rates at 71.5 ± 2.5% (VaBGs group in mice), 60 ± 2% (FKC group in mice), and 72.6 ± 2.2% (VaBGs group in fish), respectively. Thereafter, the serum antibody titer and its bactericidal rate started to decrease. The agglutinating antibody level and bactericidal activity in the immune groups were significantly higher than those in the PBS group (*p *<* *.05 or .01). Except for 7 and 14 days after immunization, the agglutinating antibody level and bactericidal activity at the other time points in the VaBGs‐immunized mice were higher than those in the FKC group (*p *<* *.05 or .01). These results revealed that the VaBGs could significantly enhance the antibody response *in vivo*.

### 3.7 Proliferation activity of spleen lymphocytes

**Figure 8:**
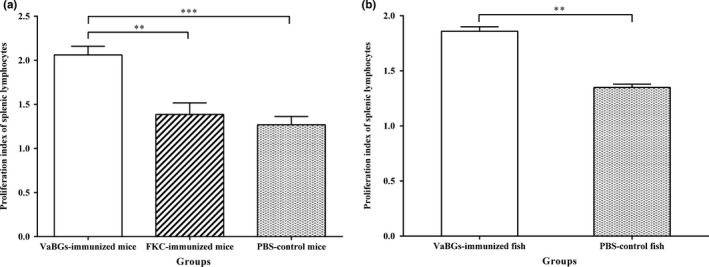
Proliferation activity of spleen lymphocytes in mice (a) and fish (b) on day 28 postimmunization. Data presented the mean ± *SD* from three experimental animals. Bars represent standard deviations. **p <* *.01, ***p <* *.001 (VaBGs group versus FKC and PBS groups)

In order to evaluate the cell‐mediated immune response, T‐lymphocyte activation after stimulation with PHA was measured by analyzing spleen lymphocytes isolated from immunized and non‐immunized animals at 4 wpi. The results showed that the average SI in the five groups were 2.06 ± 0.09 (VaBGs group in mice), 1.39 ± 0.13 (FKC group in mice), 1.27 ± 0.09 (PBS group in mice), 1.86 ± 0.04 (VaBGs group in fish), and 1.35 ± 0.03 (PBS group in fish), respectively. The T‐lymphocyte proliferative activity in the VaBGs group was very significantly higher than those in other groups (*p *<* *.01 or .001). The T‐lymphocyte proliferative activity in the FKC‐immunized mice appeared to be a little higher than that in the PBS group, but a significant difference was not reached (*p *>* *.05) (Figure 8), which indicated that the VaBGs could induce cell‐mediated immune response *in vivo*, while FKC vaccine could not.

### 3.8 Immune protection against *V. alginolyticus* challenge

**Figure 9:**
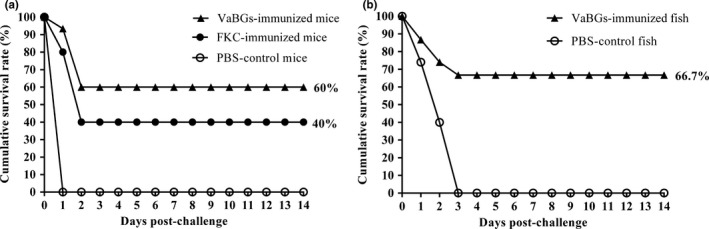
Cumulative survival rate of immunized mice (a) and fish (b) when challenged with 50 LD_50_ *Vibrio alginolyticus* strain 16‐3 on day 30 postvaccination

After the *V. alginolyticus* strain 16‐3 challenge, the challenged mice and fish showed the typical symptoms of *V. alginolyticus* infection, and the colonies of *V. alginolyticus* strain 16‐3 were recovered from all dead animals and confirmed by a PCR assay (data not shown). As shown in Figure 9, all of the mice and fish in the PBS control group died, respectively, within 1 and 3 days after challenge. The immunized mice and fish had a higher dead rate within 18–36 hr and 24–48 hr, respectively, while no death was recorded 2 or 3 days after the challenge. Compared with the control group, the relative percent survival of the VaBGs‐immunized mice, FKC‐immunized mice and VaBGs‐immunized fish was 60%, 40%, and 66.7%, respectively. These data indicated that VaBGs vaccine had higher protective efficacy than FKC vaccine.

## 4 DISCUSSION

Bacterial species identification methods mainly include phenotypic identification, such as the morphology and biochemical characteristics of bacteria, and 16S rRNA gene sequencing. Traditional biochemical tests have the disadvantages of time‐consuming and low sensitivity. Currently, some commercial bacteria identification systems, such as ATB semi‐automatic identification system (bioMerieux Sa, France), Biolog GEN III identification system (Biolog Inc., USA) and BBL Crystal E/NF identification system (Becton Dickinson Inc., USA), etc., are widely used to identify bacteria rapidly (Topic et al., 2004; Wragg, Randall, & Whatmore, 2014). However, these identification systems rely on phenotype databases. Some of these databases comprise a small repertoire of species, especially for Gram‐positive bacteria, and therefore, the results can be subjected to variability and misinterpretation. Often 16S rRNA gene sequencing is used to identify bacterial species and has been used as the reference method to compare other identification systems. But, the sequence similarity of 16S rRNA genes among different *Vibrio* species was higher, which leads to a low resolution of identification at the species level. In order to obtain accurate identification result, our research combined these two methods, and the isolated pathogen from the large yellow croaker with canker was confirmed as *V. alginolyticus* based on morphological, biochemical, and 16S rDNA sequencing results.

In recent years, outbreaks of vibriosis caused by *V. alginolyticus* have become the major cause of losses in the intensive aquaculture system. The potentiality of BGs as vaccine candidates against a wide variety of pathogenic Gram‐negative bacteria, such as *Vibrio anguillarum* (Seryun et al., 2009), *Aeromonas hydrophila* (Tu, Chu, Zhuang, & Lu, 2010), *Streptococcus iniae* (Chaehun, Sungjun, Kihong, & Sungkoo, 2010), *Flavobacterium columnare* (Zhu, Yang, Zhang, Yuan, & An, 2012) and *Edwardsiella tarda* (Wang et al., 2016), has recently been widely reported. Hence *V. alginolyticus* BGs were generated for the first time by controlled expression of the *lysis E* gene in the present study. Onset of lysis in *V. alginolyticus* occurred at 30 min and was completed by 3 hr after induction, with a lysis efficiency of 99.91%. Although *V. alginolyticus* strain 16‐3 BGs did not reach the 100% cleavage, residual live bacteria could be eliminated by lyophilization process under the condition without protective agent because intracellular water was froze into ice crystals which caused cell membrane rupture and bacterial death. In addition, animal safety testing also demonstrated that none of the immunized animals showed any adverse reactions such as abnormal behavior, mortality, or signs of *V. alginolyticus* infection. Thus, lyophilized *V. alginolyticus* 16‐3 BGs were safe and promising for use as a vaccine.

Some reports have indicated that BGs can effectively stimulate the immune system to produce humoral and cellular immune responses (Felnerova et al., 2004; Haslberger et al., 2000). To investigate the immune response following immunization with *V. alginolyticus* BGs vaccine, we firstly measured the serum agglutination antibody titer and bactericidal activity at different time points postimmunization. All of the immunized animals showed a slow increase in serum agglutination antibody titer and bactericidal activity postprimary immunization compared with the non‐immunized animals, which were dramatically enhanced after booster immunization. Results also showed both titer and bactericidal activity were significantly higher in the VaBGs group compared to the FKC group after booster immunization. In parallel, the IL‐4 level in the VaBGs group was significantly higher than that in the FKC group. IL‐4 is a Th2‐type representative cytokine which is associated with B cell proliferation, differentiation and maturity, and plays a key role in antibody production (Chen, He, Kwang, Chan, & Chen, 2012). Taken together, these results revealed that the VaBGs were more favorable to induce specific humoral immune response because the VaBGs retained all relevant antigens found on the live bacteria which can be recognized and engulfed by macrophages in immunized mice and fish.

Th1 cells are involved in the cellular immunity and secrete the effector Th1 type cytokine. So Th1 cytokine level and T‐lymphocyte proliferative activity can reflect the basic status of cellular immunity (Balenovi, Savi, Ekert, Jurinovi, & Ragland, 2011). Our data showed that, except 7 days after immunization, the Th1 type cytokine levels (IL‐2 and TNF‐α) in the VaBGs group at the other time points were significantly higher than those in the other two groups (FKC and PBS groups) (*p *<* *.05). However, there was no significant difference between the two groups (*p *>* *.05). Meanwhile, we observed markedly enhanced SI in re‐stimulated spleen T‐lymphocyte of VaBGs‐immunized mice and fish (*p *<* *.001). However, there was no significant difference between group FKC and PBS, although there was a slight increase in the SI of FKC group. These findings suggested that the *V. alginolyticus* BGs vaccine can induce immunized animals to produce a high level of cellular immune response. No cellular immune response to FKC vaccine might be related to the destruction of bacterial antigenic epitopes during the formalin‐killing process.

The RPS is an important index to evaluate the immune effect of vaccines. It has been previously demonstrated that BGs can induce a good protective efficacy against infection by wild‐type strain (Chaudhari, Jawale, Woong, & Hwa, 2012; Kwon, Lee, Nam, Kim, & Kim, 2007; Peng et al., 2011). Similar to previous findings, our results indicated that the mice and fish vaccinated with VaBGs were protected against 50 LD_50_ *V. alginolyticus* strain 16‐3 challenge, and the RPS was respectively 60% and 66.7%, which was higher than that in the FKC immunization group (40%). However, further large‐scale field trials are still needed to examine extensively the efficacy of the VaBGs vaccine.

## 5 CONCLUSIONS

This is the first report to develop *V. alginolyticus* strain 16‐3 BGs on the basis of the strain identification. The VaBGs vaccine could induce stronger cellular and humoral immune responses as well as the secretion of IL‐2, TNF‐ α, IL‐4, and IL‐6, which protect mice and fish from *V. alginolyticus* challenge as compared with conventional FKC vaccine.

## CONFLICTS OF INTEREST

The authors have no conflict of interest.
